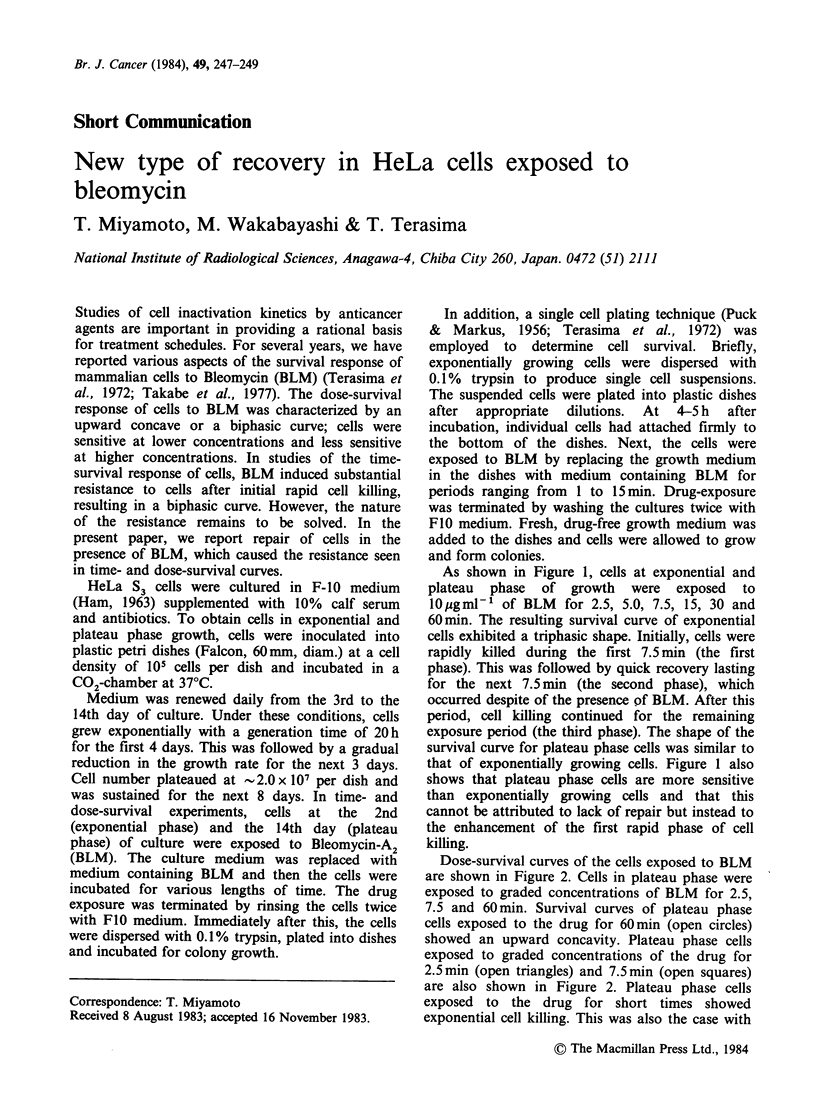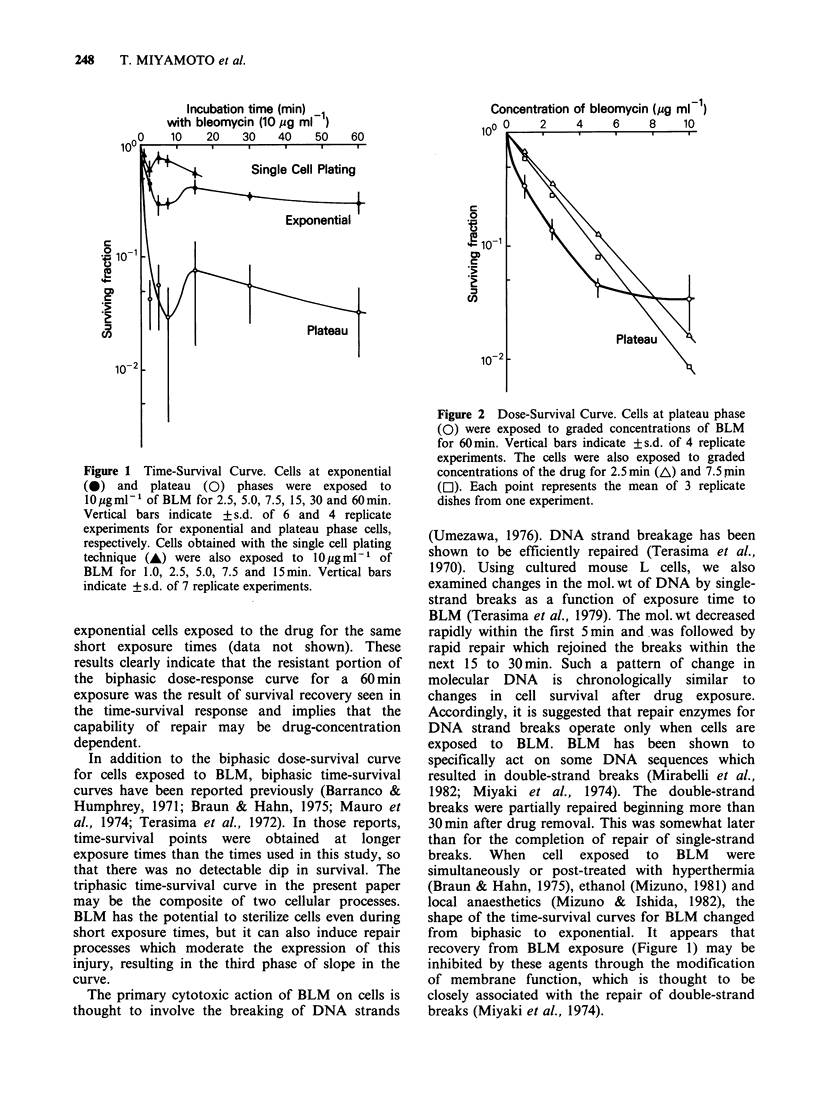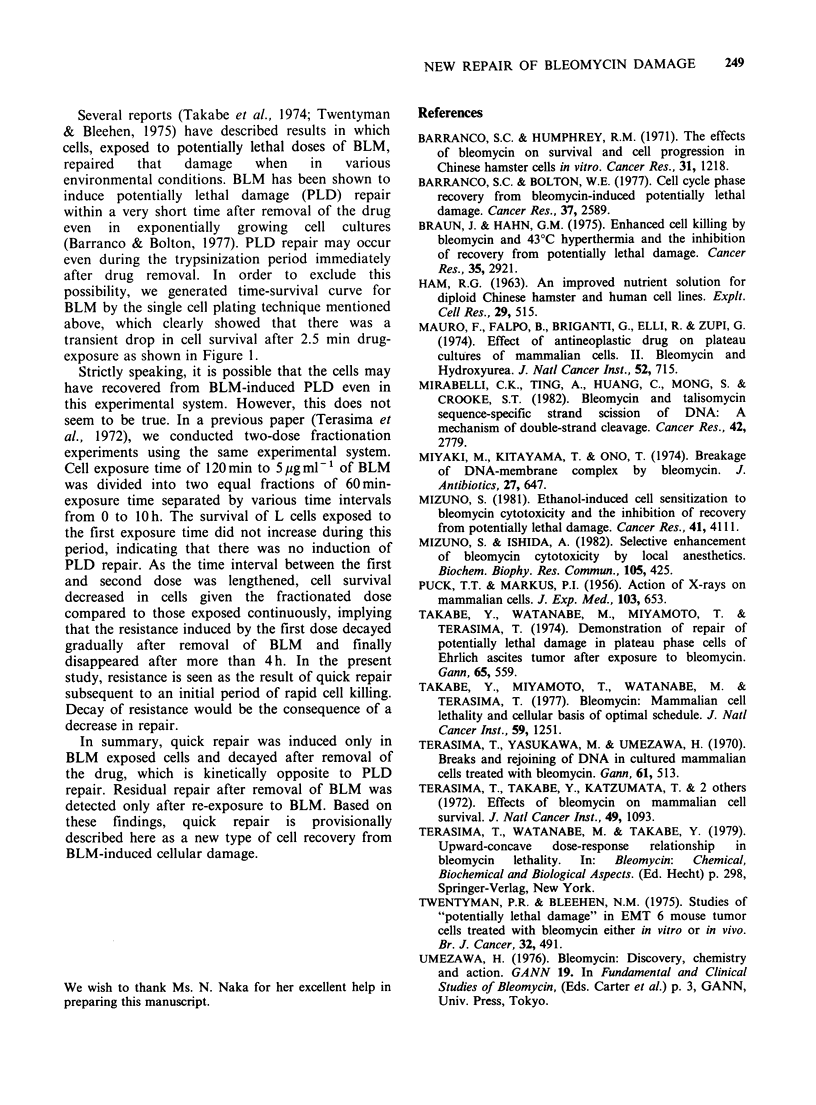# New type of recovery in HeLa cells exposed to bleomycin.

**DOI:** 10.1038/bjc.1984.39

**Published:** 1984-02

**Authors:** T. Miyamoto, M. Wakabayashi, T. Terasima


Br. J. Cancer (1984), 49, 247–249

### Short Communication

### New type of recovery in HeLa cells exposed to bleomycin

T. Miyamoto, M. Wakabayashi & T. Terasima

*National Institute of Radiological Sciences, Anagawa-4, Chiba City 260, Japan. 0472 (51) 2111*

Studies of cell inactivation kinetics by anticancer agents are important in providing a rational basis for treatment schedules. For several years, we have reported various aspects of the survival response of mammalian cells to Bleomycin (BLM) (Terasima *et al.*, 1972; Takabe *et al.*, 1977). The dose-survival response of cells to BLM was characterized by an upward concave or a biphasic curve; cells were sensitive at lower concentrations and less sensitive at higher concentrations. In studies of the time-survival response of cells, BLM induced substantial resistance to cells after initial rapid cell killing, resulting in a biphasic curve. However, the nature of the resistance remains to be solved. In the present paper, we report repair of cells in the presence of BLM, which caused the resistance seen in time- and dose-survival curves.

HeLa $S_3$ cells were cultured in F-10 medium (Ham, 1963) supplemented with 10% calf serum and antibiotics. To obtain cells in exponential and plateau phase growth, cells were inoculated into plastic petri dishes (Falcon, 60 mm, diam.) at a cell density of $10^5$ cells per dish and incubated in a $CO_2$-chamber at 37°C.

Medium was renewed daily from the 3rd to the 14th day of culture. Under these conditions, cells grew exponentially with a generation time of 20 h for the first 4 days. This was followed by a gradual reduction in the growth rate for the next 3 days. Cell number plateaued at $\sim 2.0 \times 10^7$ per dish and was sustained for the next 8 days. In time- and dose-survival experiments, cells at the 2nd (exponential phase) and the 14th day (plateau phase) of culture were exposed to Bleomycin-$A_2$ (BLM). The culture medium was replaced with medium containing BLM and then the cells were incubated for various lengths of time. The drug exposure was terminated by rinsing the cells twice with F10 medium. Immediately after this, the cells were dispersed with 0.1% trypsin, plated into dishes and incubated for colony growth.

Correspondence: T. Miyamoto
Received 8 August 1983; accepted 16 November 1983.

In addition, a single cell plating technique (Puck & Markus, 1956; Terasima *et al.*, 1972) was employed to determine cell survival. Briefly, exponentially growing cells were dispersed with 0.1% trypsin to produce single cell suspensions. The suspended cells were plated into plastic dishes after appropriate dilutions. At 4–5 h after incubation, individual cells had attached firmly to the bottom of the dishes. Next, the cells were exposed to BLM by replacing the growth medium in the dishes with medium containing BLM for periods ranging from 1 to 15 min. Drug-exposure was terminated by washing the cultures twice with F10 medium. Fresh, drug-free growth medium was added to the dishes and cells were allowed to grow and form colonies.

As shown in Figure 1, cells at exponential and plateau phase of growth were exposed to $10\,\mu g\,ml^{-1}$ of BLM for 2.5, 5.0, 7.5, 15, 30 and 60 min. The resulting survival curve of exponential cells exhibited a triphasic shape. Initially, cells were rapidly killed during the first 7.5 min (the first phase). This was followed by quick recovery lasting for the next 7.5 min (the second phase), which occurred despite of the presence of BLM. After this period, cell killing continued for the remaining exposure period (the third phase). The shape of the survival curve for plateau phase cells was similar to that of exponentially growing cells. Figure 1 also shows that plateau phase cells are more sensitive than exponentially growing cells and that this cannot be attributed to lack of repair but instead to the enhancement of the first rapid phase of cell killing.

Dose-survival curves of the cells exposed to BLM are shown in Figure 2. Cells in plateau phase were exposed to graded concentrations of BLM for 2.5, 7.5 and 60 min. Survival curves of plateau phase cells exposed to the drug for 60 min (open circles) showed an upward concavity. Plateau phase cells exposed to graded concentrations of the drug for 2.5 min (open triangles) and 7.5 min (open squares) are also shown in Figure 2. Plateau phase cells exposed to the drug for short times showed exponential cell killing. This was also the case with

© The Macmillan Press Ltd., 1984

**Figure 1** Time-Survival Curve. Cells at exponential (●) and plateau (○) phases were exposed to $10\,\mu g\,ml^{-1}$ of BLM for 2.5, 5.0, 7.5, 15, 30 and 60 min. Vertical bars indicate ±s.d. of 6 and 4 replicate experiments for exponential and plateau phase cells, respectively. Cells obtained with the single cell plating technique (▲) were also exposed to $10\,\mu g\,ml^{-1}$ of BLM for 1.0, 2.5, 5.0, 7.5 and 15 min. Vertical bars indicate ±s.d. of 7 replicate experiments.

**Figure 2** Dose-Survival Curve. Cells at plateau phase (○) were exposed to graded concentrations of BLM for 60 min. Vertical bars indicate ±s.d. of 4 replicate experiments. The cells were also exposed to graded concentrations of the drug for 2.5 min (△) and 7.5 min (□). Each point represents the mean of 3 replicate dishes from one experiment.

exponential cells exposed to the drug for the same short exposure times (data not shown). These results clearly indicate that the resistant portion of the biphasic dose-response curve for a 60 min exposure was the result of survival recovery seen in the time-survival response and implies that the capability of repair may be drug-concentration dependent.

In addition to the biphasic dose-survival curve for cells exposed to BLM, biphasic time-survival curves have been reported previously (Barranco & Humphrey, 1971; Braun & Hahn, 1975; Mauro *et al.*, 1974; Terasima *et al.*, 1972). In those reports, time-survival points were obtained at longer exposure times than the times used in this study, so that there was no detectable dip in survival. The triphasic time-survival curve in the present paper may be the composite of two cellular processes. BLM has the potential to sterilize cells even during short exposure times, but it can also induce repair processes which moderate the expression of this injury, resulting in the third phase of slope in the curve.

The primary cytotoxic action of BLM on cells is thought to involve the breaking of DNA strands (Umezawa, 1976). DNA strand breakage has been shown to be efficiently repaired (Terasima *et al.*, 1970). Using cultured mouse L cells, we also examined changes in the mol. wt of DNA by single-strand breaks as a function of exposure time to BLM (Terasima *et al.*, 1979). The mol. wt decreased rapidly within the first 5 min and was followed by rapid repair which rejoined the breaks within the next 15 to 30 min. Such a pattern of change in molecular DNA is chronologically similar to changes in cell survival after drug exposure. Accordingly, it is suggested that repair enzymes for DNA strand breaks operate only when cells are exposed to BLM. BLM has been shown to specifically act on some DNA sequences which resulted in double-strand breaks (Mirabelli *et al.*, 1982; Miyaki *et al.*, 1974). The double-strand breaks were partially repaired beginning more than 30 min after drug removal. This was somewhat later than for the completion of repair of single-strand breaks. When cell exposed to BLM were simultaneously or post-treated with hyperthermia (Braun & Hahn, 1975), ethanol (Mizuno, 1981) and local anaesthetics (Mizuno & Ishida, 1982), the shape of the time-survival curves for BLM changed from biphasic to exponential. It appears that recovery from BLM exposure (Figure 1) may be inhibited by these agents through the modification of membrane function, which is thought to be closely associated with the repair of double-strand breaks (Miyaki *et al.*, 1974).

Several reports (Takabe *et al.*, 1974; Twentyman & Bleehen, 1975) have described results in which cells, exposed to potentially lethal doses of BLM, repaired that damage when in various environmental conditions. BLM has been shown to induce potentially lethal damage (PLD) repair within a very short time after removal of the drug even in exponentially growing cell cultures (Barranco & Bolton, 1977). PLD repair may occur even during the trypsinization period immediately after drug removal. In order to exclude this possibility, we generated time-survival curve for BLM by the single cell plating technique mentioned above, which clearly showed that there was a transient drop in cell survival after 2.5 min drug-exposure as shown in Figure 1.

Strictly speaking, it is possible that the cells may have recovered from BLM-induced PLD even in this experimental system. However, this does not seem to be true. In a previous paper (Terasima *et al.*, 1972), we conducted two-dose fractionation experiments using the same experimental system. Cell exposure time of 120 min to 5 $\mu g\, ml^{-1}$ of BLM was divided into two equal fractions of 60 min-exposure time separated by various time intervals from 0 to 10 h. The survival of L cells exposed to the first exposure time did not increase during this period, indicating that there was no induction of PLD repair. As the time interval between the first and second dose was lengthened, cell survival decreased in cells given the fractionated dose compared to those exposed continuously, implying that the resistance induced by the first dose decayed gradually after removal of BLM and finally disappeared after more than 4 h. In the present study, resistance is seen as the result of quick repair subsequent to an initial period of rapid cell killing. Decay of resistance would be the consequence of a decrease in repair.

In summary, quick repair was induced only in BLM exposed cells and decayed after removal of the drug, which is kinetically opposite to PLD repair. Residual repair after removal of BLM was detected only after re-exposure to BLM. Based on these findings, quick repair is provisionally described here as a new type of cell recovery from BLM-induced cellular damage.

We wish to thank Ms. N. Naka for her excellent help in preparing this manuscript.

### References

BARRANCO, S.C. & HUMPHREY, R.M. (1971). The effects of bleomycin on survival and cell progression in Chinese hamster cells *in vitro*. *Cancer Res.*, **31**, 1218.

BARRANCO, S.C. & BOLTON, W.E. (1977). Cell cycle phase recovery from bleomycin-induced potentially lethal damage. *Cancer Res.*, **37**, 2589.

BRAUN, J. & HAHN, G.M. (1975). Enhanced cell killing by bleomycin and 43°C hyperthermia and the inhibition of recovery from potentially lethal damage. *Cancer Res.*, **35**, 2921.

HAM, R.G. (1963). An improved nutrient solution for diploid Chinese hamster and human cell lines. *Explt. Cell Res.*, **29**, 515.

MAURO, F., FALPO, B., BRIGANTI, G., ELLI, R. & ZUPI, G. (1974). Effect of antineoplastic drug on plateau cultures of mammalian cells. II. Bleomycin and Hydroxyurea. *J. Natl Cancer Inst.*, **52**, 715.

MIRABELLI, C.K., TING, A., HUANG, C., MONG, S. & CROOKE, S.T. (1982). Bleomycin and talisomycin sequence-specific strand scission of DNA: A mechanism of double-strand cleavage. *Cancer Res.*, **42**, 2779.

MIYAKI, M., KITAYAMA, T. & ONO, T. (1974). Breakage of DNA-membrane complex by bleomycin. *J. Antibiotics*, **27**, 647.

MIZUNO, S. (1981). Ethanol-induced cell sensitization to bleomycin cytotoxicity and the inhibition of recovery from potentially lethal damage. *Cancer Res.*, **41**, 4111.

MIZUNO, S. & ISHIDA, A. (1982). Selective enhancement of bleomycin cytotoxicity by local anesthetics. *Biochem. Biophy. Res. Commun.*, **105**, 425.

PUCK, T.T. & MARKUS, P.I. (1956). Action of X-rays on mammalian cells. *J. Exp. Med.*, **103**, 653.

TAKABE, Y., WATANABE, M., MIYAMOTO, T. & TERASIMA, T. (1974). Demonstration of repair of potentially lethal damage in plateau phase cells of Ehrlich ascites tumor after exposure to bleomycin. *Gann*, **65**, 559.

TAKABE, Y., MIYAMOTO, T., WATANABE, M. & TERASIMA, T. (1977). Bleomycin: Mammalian cell lethality and cellular basis of optimal schedule. *J. Natl Cancer Inst.*, **59**, 1251.

TERASIMA, T., YASUKAWA, M. & UMEZAWA, H. (1970). Breaks and rejoining of DNA in cultured mammalian cells treated with bleomycin. *Gann*, **61**, 513.

TERASIMA, T., TAKABE, Y., KATZUMATA, T. & 2 others (1972). Effects of bleomycin on mammalian cell survival. *J. Natl Cancer Inst.*, **49**, 1093.

TERASIMA, T., WATANABE, M. & TAKABE, Y. (1979). Upward-concave dose-response relationship in bleomycin lethality. In: *Bleomycin: Chemical, Biochemical and Biological Aspects*. (Ed. Hecht) p. 298, Springer-Verlag, New York.

TWENTYMAN, P.R. & BLEEHEN, N.M. (1975). Studies of "potentially lethal damage" in EMT 6 mouse tumor cells treated with bleomycin either *in vitro* or *in vivo*. *Br. J. Cancer*, **32**, 491.

UMEZAWA, H. (1976). Bleomycin: Discovery, chemistry and action. *GANN* **19**. In *Fundamental and Clinical Studies of Bleomycin*, (Eds. Carter *et al.*) p. 3, GANN, Univ. Press, Tokyo.